# Identification of serum angiopoietin-2 as a biomarker for clinical outcome of colorectal cancer patients treated with bevacizumab-containing therapy

**DOI:** 10.1038/sj.bjc.6605925

**Published:** 2010-10-05

**Authors:** V Goede, O Coutelle, J Neuneier, A Reinacher-Schick, R Schnell, T C Koslowsky, M R Weihrauch, B Cremer, H Kashkar, M Odenthal, H G Augustin, W Schmiegel, M Hallek, U T Hacker

**Affiliations:** 1 Department of Internal Medicine I, Center of Integrated Oncology Cologne-Bonn, University Hospital Cologne, Kerpener Straße 62, Cologne 50924, Germany; 2 Department of Internal Medicine, University Hospital Bochum, In der Schornau 23-25, Bochum 44892, Germany; 3 Oncology and Hematology Practice, Kölner Strasse 9, Frechen 50226, Germany; 4 Department of Surgery, St Elisabeth Hospital, Werthmannstr. 1, Cologne 50935, Germany; 5 Institute for Medical Microbiology, Immunology and Hygiene, University of Cologne, Goldenfels Strasse 19-21, Cologne 50935, Germany; 6 Department of Pathology, University of Cologne, Kerpener Straße 62, Cologne 50924, Germany; 7 Joint Research Division Vascular Biology, Medical Faculty Mannheim (CBTM), Heidelberg University and German Cancer Research Center Heidelberg (DKFZ-ZMBG Alliance), Im Neuenheimer Feld 280, Heidelberg 69120, Germany

**Keywords:** colorectal cancer, angiopoietin-2, biomarker, chemotherapy, bevacizumab

## Abstract

**Background:**

The combination of chemotherapy with the vascular endothelial growth factor (VEGF) antibody bevacizumab is a standard of care in advanced colorectal cancer (CRC). However, biomarkers predicting outcome of bevacizumab-containing treatment are lacking. As angiopoietin-2 (Ang-2) is a key regulator of vascular remodelling in concert with VEGF, we investigated its role as a biomarker in metastatic CRC.

**Methods:**

Serum Ang-2 levels were measured in 33 healthy volunteers and 90 patients with CRC. Of these, 34 had metastatic disease and received bevacizumab-containing therapy. To determine the tissue of origin of Ang-2, quantitative real-time PCR was performed on microdissected cryosections of human CRC and in a murine xenograft model of CRC using species-specific amplification.

**Results:**

Ang-2 originated from the stromal compartment of CRC tissues. Serum Ang-2 levels were significantly elevated in patients with metastatic CRC compared with healthy controls. Amongst patients receiving bevacizumab-containing treatment, low pre-therapeutic serum Ang-2 levels were associated with a significant better response rate (82 *vs* 31% *P*<0.01), a prolonged median progression-free survival (14.1 *vs* 8.5 months; *P*<0.01) and a reduction of 91% in the hazard of death (*P*<0.05).

**Conclusion:**

Serum Ang-2 is a candidate biomarker for outcome of patients with metastatic CRC treated with bevacizumab-containing therapy, and it should be further validated to customise combined chemotherapeutic and anti-angiogenic treatment.

The overall survival (OS) time of patients with metastatic colorectal cancer (CRC) has increased owing to novel therapeutic strategies combining chemotherapy with monoclonal antibodies against vascular endothelial growth factor (VEGF) or epidermal growth factor receptor (EGFR). Whereas mutations in the *k-ras* oncogene predict outcome to EGFR antibody treatment in patients with metastatic CRC (Lievre *et al*, 2006; Karapetis *et al*, 2008), equivalent biomarkers for the VEGF antibody, bevacizumab, are currently lacking (Jubb *et al*, 2006b; Sessa *et al*, 2008), mainly because the specific molecular determinants of clinical response and resistance to the drug are unknown. Similarly, there are currently no established biomarkers predicting outcome to chemotherapy in CRC (De Roock *et al*, 2009).

Originally, it was anticipated that traditional markers of tumour angiogenesis would predict outcome to bevacizumab. However, neither VEGF expression levels nor tumour microvessel density (MVD) were found to be predictive of treatment response, disease progression or death in CRC patients receiving chemotherapy plus the antibody (Jubb *et al*, 2006a). Newer studies evaluated alternative surrogates of neovascularisation such as circulating endothelial cells and phosphorylated VEGF receptor in cancers other than CRC (Murukesh *et al*, 2010). Results were promising, but assay requirements prevent widespread application in larger trials.

The therapeutic blockade of VEGF by bevacizumab in CRC patients induces complex changes in the stromal compartment of the tumour lesion, including the loss of chaotic microvessels, remodelling of the vascular wall and a reduction in the interstitial fluid pressure (Willett *et al*, 2004). Such stromal alterations are part of the vascular ‘normalisation’ process induced by bevacizumab and contribute to more efficient delivery of chemotherapeutic agents (Jain, 2005; Jain *et al*, 2006). Recently, dynamic contrast-enhanced magnetic resonance imaging was proposed to assess the extent of vascular normalisation and predict clinical outcome to VEGF-targeting treatment (Sorensen *et al*, 2009; Murukesh *et al*, 2010). However, molecular markers reflecting the normalisation status of the tumour vascular bed as part of the tumour stroma have not been explored in this regard so far.

The molecular alterations of tumour cells are traditionally regarded as the major determinants of clinical response to chemotherapy. However, the above observations suggest that the vascular microenvironment of tumour cells could be equally important for efficacious cytostatic treatment. Surrogates of vascular normalisation, therefore, might predict outcome to chemotherapy as well as to VEGF-targeting therapy.

Angiopoietin-2 (Ang-2) is an inhibitory ligand of the Tie-2 receptor that is stored in the Weibel–Palade bodies of endothelial cells (Fiedler *et al*, 2004) and disrupts the integrity of the blood vessel wall, thus counteracting vascular normalisation (Maisonpierre *et al*, 1997; Scharpfenecker *et al*, 2005; Falcon *et al*, 2009; Reiss *et al*, 2009). On the basis of this, we expected Ang-2 to be predominantly expressed in the stromal compartment of CRC, and hypothesised that low and high serum Ang-2 levels in patients with metastatic CRC would predict different outcomes to bevacizumab-containing treatment.

## Materials and methods

### Microdissection

For microdissection analysis, cyrosections were prepared from human adenocarcinoma samples. Discrete areas of tumour or stromal tissue were microdissected using a laser microbeam (P.A.L.M., Bernried, Germany). Microdissected tissue areas (each 100 *μ*m in diameter, 30 per slide for stroma and tumour each) were laser catapulted into a microfuge tube.

### Xenografts

Animal experiments were performed in accordance with the German animal protection law. The colon carcinoma cell line LS174T was purchased from ATCC (Manassas, VA, USA) to establish xenografts in nude mice. Cultured LS174T cells (5 × 10^6^) were injected subcutaneously into the flank region of male BALB/cA nude mice (Taconic, Ry, Denmark). Animals were killed after tumour size had reached 10 mm in diameter.

### Cell cultures

The colon carcinoma cell lines LS174T, HT29, DLD-1 and SW948 were cultured in VLE RPMI 1640 (Biochrom, Berlin, Germany) supplemented with 10% FCS. Human umbilical vein endothelial cells were purchased from Promocell (Heidelberg, Germany) and cultured in endothelial cell growth medium (Promocell). All cell lines were maintained at 37°C and 5% CO_2_, except for one experiment involving exposure to controlled hypoxia (1% O_2_, 5% CO_2_, balanced N_2_) for 24 h.

### Quantitative real-time PCR

The RNA was extracted from tissue microdissections using the NucleoSpin RNA isolation kit (Macherey-Nagel, Düren, Germany) and reverse transcribed using the RevertAid cDNA Synthesis Kit (Fermentas, St Leon-Rot, Germany). For gene expression analysis in xenografts, tumours were suspended in RNAlater (Qiagen, Hilden, Germany). The RNA was extracted as described above. Real-time PCR for Ang-2 was carried out using the LightCycler FastStart DNA Master Plus Mix (Roche, Basel, Switzerland). Results were normalised to a dilution series of pre-quantified pooled PCR products with the RelQuant Software (Roche). Primer sequences for human- and murine-specific amplification of Ang-2 and *β*-actin were as published (Thijssen *et al*, 2004). Universal GAPDH primers were as follows: Forward 5′-TGC(A/C)TCCTGCACCACCAACT-3′, Reverse 5′(C/T)GCCTGCTTCACCACCTTC-3′.

### Western blot

For western blot (WB) analysis, cytosolic extracts of cultured cells were prepared as described previously (Kashkar *et al*, 2002). Equal amounts (100 *μ*g) of protein were separated by SDS-PAGE, transferred to a nitrocellulose membrane (Schleicher & Schuell, Dassel, Germany), and probed with Ang-2 antibody (F18, Santa Cruz Biotechnology, Santa Cruz, CA, USA). Primary antibodies were detected using a horseradish-peroxidase-conjugated secondary antibody (1 : 2000; Dako, Hamburg, Germany) and visualised with the ECL system (Amersham Biosciences, Hamburg, Germany). Culture supernatants were analysed for Ang-2 protein concentrations using Quantikine Immunoassays (R&D Systems, Wiesbaden, Germany).

### Clinical samples

A total of 90 patients with colorectal adenocarcinoma and 33 healthy volunteers were studied between September 2005 and November 2008. One cohort of 56 patients had newly diagnosed CRC of various stages (UICC I–IV). After obtaining informed consent, serum samples and tumour tissues were collected at the time of primary resection (sampling from September 2005 to August 2006). A second cohort of 34 patients had primary (*n*=25) or relapsed (*n*=9) CRC of advanced stage and received a combination treatment of bevacizumab and chemotherapy either in the context of a clinical trial (AIO trial KRK 0604, *n*=15) conducted by the Department of Internal Medicine of the University Hospital Bochum (Reinacher-Schick *et al*, 2008) or without participating in a clinical trial (*n*=19) at the Center of Integrated Oncology Cologne-Bonn. Following approval by the Ethics Committees, serum samples from these patients were taken prior to treatment (sampling from March 2006 to April 2008). Where available, paraffin blocks of tumour tissue were retrieved from the local pathology archives.

Demographical, clinical and histopathological baseline parameters were documented in all patients. For patients receiving bevacizumab-containing therapy, the clinical response after 2 months of treatment was assessed according to response evaluation criteria in solid tumours (RECIST). Patients were continuously monitored during the course of treatment and disease progression and deaths occurring during and after therapy were recorded.

### Enzyme-linked immunosorbent assay

Quantikine Immunoassays (R&D Systems) were used to measure protein concentrations of Ang-2 and VEGF in serum samples according to the manufacturer's instructions.

### Immunohistochemistry

Sections of paraffin tissue blocks of CRC specimens were processed for histological analysis as previously described (Eberhard *et al*, 2000). Immunohistochemistry (IHC) for Ang-2 was carried out with the following antibodies: MAB 0983 (R&D Systems), N18 and F18 (Santa Cruz Biotechnology). To assess non-specific antibody binding, Ang-2 antibodies were blocked by pre-incubation with recombinant Ang-2 (ratio 1 : 5, R&D systems) prior to incubation of histological sections. A biotinylated secondary antibody, streptavidin peroxidase complex, and diaminobenzidine as the substrate (Zymed, Carlsbad, CA, USA) were used to visualise binding of the primary antibody (followed by semiquantitative assessment of Ang-2 staining intensity). Tumour blood vessels were stained with the CD34 antibody QBEnd/10 (Novocastra Laboratories, Wetzlar, Germany) and the *α*-SMA antibody 1A4 (Dako). Microvessel density and pericyte coverage (PC) were assessed as described previously (Eberhard *et al*, 2000).

### Statistical analysis

The SPSS 17 software (SPSS Inc., Chicago, IL, USA) was used for data analysis. Areas under receiver operating characteristic curves (AUROCs) were measured to assess the diagnostic performance of continuous test variables for treatment response as the target variable. Youden's index was calculated to determine cutoff values of test variables. Parameters others than survival were compared using the Mann–Whitney test and the *χ*^2^-test. Overall and progression-free survival (PFS) was calculated using the Kaplan–Meier method. The log-rank test was used to compare survival time periods between groups. A Cox regression model was applied for univariate and multivariate analysis to estimate hazard ratios. All statistical tests were two sided. Statistical significance was defined as *P*<0.05. Data analysis is reported according to REMARK guidelines (Mallett *et al*, 2010).

## Results

### Ang-2 is expressed in the stromal compartment of CRC but not in the tumour cells

**Figure 1:**
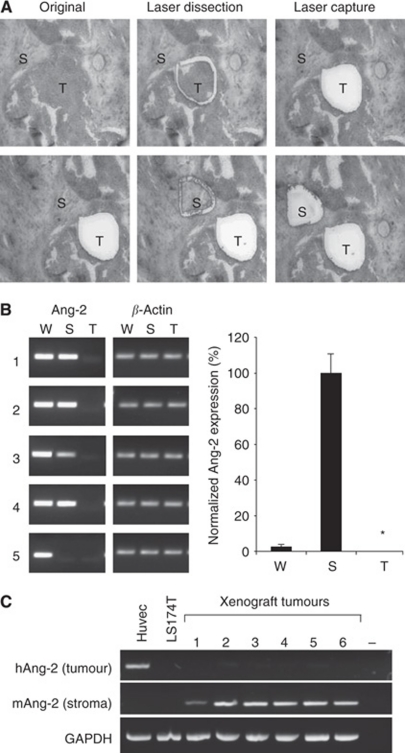
Expression of Ang-2 in CRC. (**A**) Illustration of the steps involved in laser microdissection of cryosections of CRC patients to obtain stromal (S) and tumour (T) material for RT–PCR amplification. The top row shows capture of tumour and the bottom row capture of stroma from the same section. (**B**) Gel electrophoresis of end point RT–PCR amplification products of Ang-2 and *β*-actin on material of a whole tumour section (W) or microdissected stromal or tumour areas of sections of five different CRC patients showing Ang-2 expression in W and S, but not in T. *β*-Actin served as a control. The bar chart shows the relative quantitative real-time PCR of Ang-2 levels normalised to *β*-actin expression using the Δ*C*_t_ method. Bars represent the means of five patients with standard errors showing significant Ang-2 expression (100%) in the tumour stroma, but undetectable in the tumour cells themselves (^*^). Some expression (<10%) is also seen when whole sections were amplified, owing to the Ang-2 expressing stromal compartment. (**C**) End point RT–PCR analysis of xenograft tumours of human LS174T cells in nude mice analysed by species-specific amplification for human (tumour) and murine (stromal) Ang-2. Xenograft tumours show stromal-derived murine Ang-2, but not tumour-derived human Ang-2. The GAPDH was used as a species independent control. Human umbilical vein endothelial cells (HUVEC) served as a positive control for human Ang-2.

Extensive immunohistochemical analysis of Ang-2 with three frequently reported commercial Ang-2 antibodies produced ambiguous results concerning the tissue localisation in human CRC because of poor antibody specificity (Supplementary Figure 1). Therefore, to identify the tissue of origin of Ang-2 in CRC, laser-captured microdissection was used to isolate the tumour and stromal compartments from tissue sections of CRC patients for quantitative real-time PCR (Figure 1A). The Ang-2 mRNA was clearly detectable in the dissected stromal compartment, but not in the tumour cell compartment (Figure 1B).

Xenografts of CRC in nude mice were generated to further verify the stromal origin of Ang-2. In these animals, the stromal compartment is of murine origin and the tumour cell compartment is of human origin (LS174T colon carcinoma cells). Using species-specific RT–PCR, Ang-2 mRNA expression was found to be exclusively of stromal (murine) origin (Figure 1C). Correspondingly, no significant amount of Ang-2 protein was detectable in cytosolic extracts and culture supernatants of various colon carcinoma cell lines (LS174T, HT29, DLD-1 and SW948) cultured under normoxia or hypoxia to mimic the conditions inside the tumour (Supplementary Figure 2).

### Serum Ang-2 levels are elevated in CRC patients with metastatic disease

**Figure 2:**
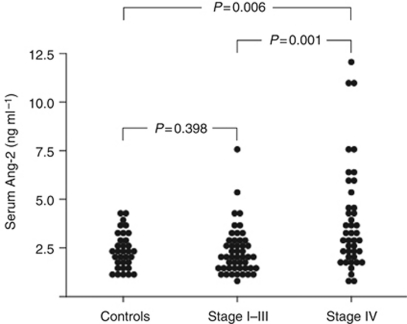
Serum levels of Ang-2 in CRC (*n*=90). Serum Ang-2 in healthy controls, non-metastatic (stage I–III) and metastatic disease (stage IV). Ang-2, angiopoietin-2.

Blood serum concentrations of Ang-2 were studied in a total of 90 patients with colorectal adenocarcinoma and 33 healthy volunteers. The patient demographics are summarised in Table 1. In UICC stage IV patients, serum Ang-2 levels were significantly higher compared with patients with UICC stage I–III or healthy controls (3.9 *vs* 2.3 ng ml^−1^, *P*=0.001 and 3.9 *vs* 2.4 ng ml^−1^, *P*=0.006, respectively) (Figure 2). In contrast, serum Ang-2 levels did not differ significantly between UICC stage I–III patients and controls.

### Serum Ang-2 levels identify CRC patients of different clinical outcomes to bevacizumab-containing therapy

On enrolment, 34 patients with primary or relapsed CRC of UICC stage IV were treated with bevacizumab in combination with chemotherapy and followed for clinical outcome. Individual patient characteristics and outcomes are presented in Table 2. A total of 19 patients responded to treatment, whereas the rest had stable or progressive disease. The overall PFS was 12.1 months. After a median follow-up period of 16.6 months (range 4–26), the median OS had not been reached. No significant differences in survival times and response rates were found between different study sites, gender or age groups (Supplementary Table 1).

Pre-therapeutic serum Ang-2 concentrations in CRC patients ranged from 0.7 to 12.1 ng ml^−1^ (median: 3.5 ng ml^−1^) and did not correlate with other vascular markers such as serum levels of VEGF (median: 0.19 ng ml^−1^), tumour MVD (median: 42 per HPF) or PC (median: 52%). Measured serum Ang-2 and VEGF levels were unbiased by gender, age, ECOG status, treatment line or regimen and showed no correlation with the numbers of metastatic sites (Supplementary Table 2).

**Figure 3:**
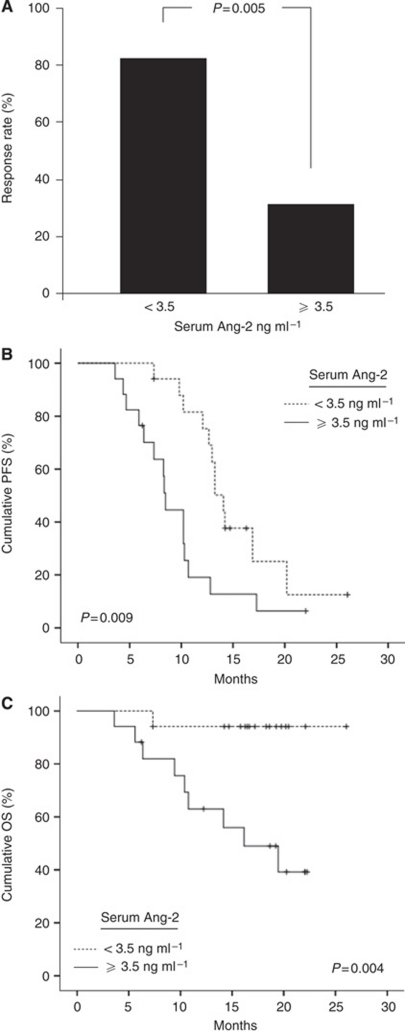
Outcome to bevacizumab-containing therapy in CRC by serum Ang-2 (*n*=34). (**A**) Response by serum Ang-2. (**B**) PFS by serum Ang-2. (**C**) OS by serum Ang-2. Ang-2, angiopoietin-2.

**Figure 4:**
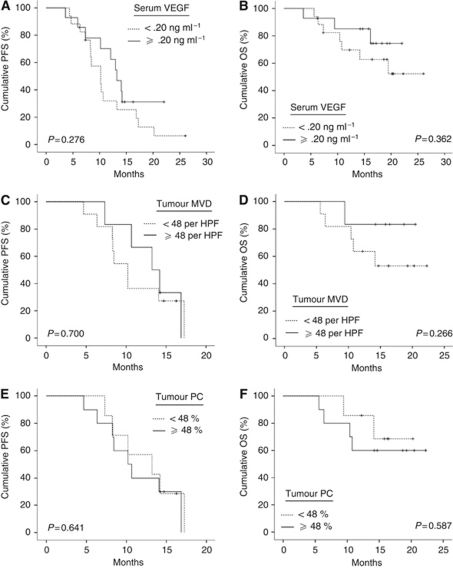
Outcome to bevacizumab-containing therapy in CRC by serum VEGF (*n*=34), tumour MVD and PC (*n*=17). (**A**) PFS by serum VEGF. (**B**) OS by serum VEGF. (**C**) PFS by MVD. (**D**) OS by MVD. (**E**) PFS by PC. (**F**) OS by PC. VEGF, vascular endothelial growth factor, MVD, microvessel density, PC, pericyte coverage.

The AUROC was 0.77 for serum Ang-2 (95% CI: 0.61–0.94; *P*=0.008) when using treatment response as the outcome parameter. The AUROC for tumour MVD was 0.81, but statistical significance was marginal (95% CI: 0.53–1.00; *P*=0.03). For serum VEGF and PC, AUROC did not differ significantly from 0.50. Based on ROC analysis and Youden's index, a serum Ang-2 of 3.5 ng ml^−1^ was chosen as the cutoff value and patients were dichotomised into subgroups with low or high serum Ang-2 concentrations. Significantly more patients with low serum Ang-2 levels responded to treatment than patients with high serum Ang-2. The overall response rate (OR) in the two groups was 82 and 31% (*P*=0.005), respectively (Figure 3A). Mean serum Ang-2 concentrations were lower in treatment responders compared with non-responders (3.3 *vs* 5.8 ng ml^−1^; *P*=0.008). Disease control was significantly better in patients with low serum Ang-2 levels than in patients with high serum Ang-2 (median PFS: 14.1 *vs* 8.5 months; *P*=0.009) (Figure 3B). There was a 63% reduction in the hazard of progression for patients with low serum Ang-2 compared with those with high serum Ang-2 (HR 0.37; 95% CI: 0.17–0.80; *P*=0.01). Overall survival was remarkably prolonged in patients with low serum Ang-2 levels (median OS: not reached) compared with the group of patients with high serum Ang-2 (median OS: 16.2 months; *P*=0.004). Survival rates at 1.5 years were 94 *versus* 53% in the low and high serum Ang-2 group, respectively (Figure 3C), and the hazard of death in low Ang-2 patients was reduced by 91% compared with patients with high serum Ang-2 levels (HR 0.09; 95% CI: 0.01–0.70; *P*=0.02). Differences in survival remained significant when patients with primary and relapsed CRC were analysed separately (Supplementary Table 3), and in a multivariate analysis of variables with potential impact on OR, PFS and OS (gender, age, site, treatment regimen and treatment line), serum Ang-2 was confirmed as an independent prognostic marker for all three end points (*P*=0.003, *P*=0.005 and *P*=0.003, respectively). In contrast to serum Ang-2 levels, there was no significant association between OR, PFS or OS and low *vs* high serum VEGF or tumour PC, respectively, using cutoff values as determined by ROC analysis and Youden's index. Similarly, tumour MVD was not associated with these end points except for OR (Table 2, Figure 4).

## Discussion

Bevacizumab is a VEGF-targeting antibody that is widely used in combination with chemotherapy to treat metastatic CRC (Cercek and Saltz, 2008; McCormack and Keam, 2008). Although much has been learned about its mechanisms of action, suitable biomarkers predicting patients who are likely to benefit from bevacizumab treatment remain elusive (Jubb *et al*, 2006b; Sessa *et al*, 2008; Murukesh *et al*, 2010). Expression levels of VEGF, in particular, are not predictive of outcome in CRC patients treated with bevacizumab (Jubb *et al*, 2006a). So far, the search for outcome predictors in CRC has failed for bevacizumab and antibody-free chemotherapeutic regimens alike (De Roock *et al*, 2009).

Although VEGF is primarily produced by tumour cells, its target structure is the tumour vasculature embedded in the stromal compartment, where the therapeutic effects of the antibody involve extensive changes such as blood vessel pruning and reorganisation of the chaotic tumour vasculature (Willett *et al*, 2004). Thus, stromal factors controlling the responsiveness of blood vessels to VEGF withdrawal rather than determinants of VEGF availability are attractive candidates as outcome predictors for bevacizumab treatment. Potentially, stromal factors are also outcome predictors of chemotherapy, because the delivery of cytostatic drugs to tumour cells is controlled by the vascular tumour microenvironment.

Angiopoietin-2 has been proposed as a gatekeeper of VEGF function and vascular remodelling. (Hanahan, 1997; Augustin *et al*, 2009). We here identified Ang-2 as a stromal factor in CRC. In tumour lesions of CRC patients and in a murine xenograft model of CRC, Ang-2 mRNA was expressed exclusively in the tumour stromal compartment, but not in the tumour cell compartment itself. Although these findings are at odds with previous immunohistochemical studies reporting Ang-2 expression in the tumour cell compartment of CRC (Ochiumi *et al*, 2004; Ogawa *et al*, 2004; Chung *et al*, 2006; Gu *et al*, 2006), the published immunohistochemical data should be interpreted with caution owing to the limited specificity of the available antibodies. Indeed, careful analysis of the tissue localisation of Ang-2 expression in cancer entities and tumour models other than CRC has called into question the tumour cell origin of Ang-2 (Zhang *et al*, 2003).

To further elucidate the clinical impact of stromal-derived Ang-2, we measured serum Ang-2 concentrations in CRC patients. Serum Ang-2 levels were significantly elevated in patients with metastatic disease. Indeed, Ang-2 has been shown to promote metastatic growth (Imanishi *et al*, 2007). Although one can only speculate as to what extent the stromal expression of Ang-2 contributes to serum Ang-2 concentrations in patients with advanced CRC, serum levels of Ang-2 in such patients were found to be significantly higher than in healthy individuals. It seems likely, therefore, that serum Ang-2 levels reflect the level of Ang-2 expression in the tumour stromal compartment.

Elevated serum concentrations of Ang-2 have also been reported for patients with cancers other than CRC, such as non-small-cell lung cancer and melanoma, in which high serum Ang-2 levels correlate with disease stage and poor OS (Park *et al*, 2007; Helfrich *et al*, 2009). However, the relationship between serum Ang-2 levels and clinical outcome in patients treated with VEGF-targeting drugs and chemotherapeutic agents has not been explored before, and this study is the first to investigate the impact of pre-therapeutic serum Ang-2 concentrations on the clinical outcome in patients with metastatic CRC under bevacizumab-containing therapy. Compared with high serum Ang-2 levels, low serum Ang-2 was associated with an outstanding response rate (>80%), better disease control and excellent OS (>90% after 18 months). In accordance with previous reports (Jubb *et al*, 2006a), VEGF and tumour MVD were not similarly correlated to these end points. Similarly, the pericyte content of CRC was not linked to treatment outcome and did not correlate with Ang-2 serum concentrations, indicating that serum Ang-2 is probably not simply a surrogate of blood vessel morphology.

On the basis of experimental models, Ang-2 has been described as an opponent of vascular normalisation that prevents blood vessels from becoming structurally and functionally stabilised (Maisonpierre *et al*, 1997; Scharpfenecker *et al*, 2005; Falcon *et al*, 2009; Reiss *et al*, 2009). Conceivably, normalisation of tumour vessels by bevacizumab-mediated blockade of VEGF may be more difficult to achieve and chemotherapeutic drugs cannot be delivered appropriately to the tumour cells when Ang-2 serum levels are high. From a mechanistic point of view, the observation that patients with low serum Ang-2 were most likely to benefit from treatment with respect to major clinical end points supports such a biological role of Ang-2. From a clinical perspective, our observations suggest that serum Ang-2 could hold promise as a predictive biomarker allowing bevacizumab-containing treatment to be customised in CRC patients.

Having analysed a heterogeneous patient cohort of moderate size, our study is not without limitations. Nevertheless, subgroup analyses of known prognostic factors in CRC (for example, age, ECOG) showed no evidence that outcome by Ang-2 was biased by those factors. The major and novel finding of this study is that pre-therapeutic serum Ang-2 not only predicted OS in the study population but was also predictive of therapeutic end points (PFS, response rate), suggesting that Ang-2 is a stromal determinant of both resistance and response to bevacizumab-containing therapy. As this is a single-arm study, it remains undecided whether serum Ang-2 is a specific outcome predictor for bevacizumab or chemotherapy or for the combination of both. Irrespective of its specific predictive properties, however, measurements of pre-therapeutic serum Ang-2 could be valuable. For example, CRC patients awaiting secondary resection of metastases could be stratified by serum Ang-2 levels into risk groups. Whereas patients with low serum Ang-2 are likely to benefit from neoadjuvant bevacizumab-containing treatment, patients with high serum Ang-2 could require escalation of chemotherapy or use of other biologicals such as EGFR antibodies in patients with wild type *k-ras* oncogene or Ang-2 inhibitors that are currently in clinical development (Hu and Cheng, 2009). Although definitive judgement on the role of Ang-2 as a specific biomarker of outcome to bevacizumab in CRC or other cancers will require analysis of large numbers of blood samples from phase III clinical trials comparing bevacizumab-containing therapy with chemotherapy alone, the promising results of this study should encourage researchers to further investigate the predictive value of Ang-2 in cancer treatment.

## Figures and Tables

**Table 1:** Demographics of studied patients

	***N* (%)**
Patients	90 (100.0)
	
*Sex*	
Male	54 (60.0)
Female	36 (40.0)
	
*Age*	
Median (years)	69
Range (years)	45–86
<65 years	31 (34.4)
⩾65 years	59 (65.6)
	
*Stage (UICC*)^a^	
I	3 (3.3)
II	30 (33.3)
III	15 (16.7)
IV	42 (46.7)
	
*Systemic therapy*	
Unfollowed^b^	56 (62.2)
Followed^c^	34 (37.8)
Controls	33

Abbreviations: BV=bevacizumab; CRC=colorectal cancer; UICC=Union for International Cancer Control.

a At enrolment.

b Patients with primary CRC who underwent tumour resection and were not followed up for outcome of any further treatment.

c Patients with primary or relapsed CRC who received BV-containing therapy and were followed up for treatment outcome.

**Table 2:** Characteristics and individual outcomes of patients with metastatic disease treated with bevacizumab-containing therapy

				**Treatment**		**Outcome**						
**Sex**	**Age (years)**	**ECOG**	**Metas. (sites)**	**Regimen**	**Line**	**Initial response**	**Time to progress (months)**	**Time to death (months)**	**Ang-2 (ng ml^−1^)**	**VEGF (ng ml^−1^)**	**MVD (per HPF)**	**PC (%)**
M	63	1	1	FOLFIRI+BV	1st	Yes	—	—	0.7	0.05	NA	NA
M	67	0	1	XELIRI+BV	1st	Yes	—	—	2.4	0.19	NA	NA
M	57	1	1	FOLFIRI+BV	2nd	Yes	—	—	2.4	0.20	29	80
M	69	1	2	FOLFIRI+BV	1st	Yes	—	—	2.9	0.25	58	56
F	69	1	1	FOLFOX+BV	1st	Yes	—	—	2.9	0.46	42	20
M	56	1	1	FOLFIRI+BV	2nd	Yes	—	—	4.1	0.31	NA	NA
M	59	1	1	XELOX+BV	1st	No	—	—	3.8	0.20	NA	NA
M	70	0	1	XELIRI+BV	1st	Yes	20	—	3.3	0.02	NA	NA
F	54	1	1	FOLFIRI+BV	2nd	Yes	17	—	2.1	0.10	53	60
F	57	1	2	FOLFIRI+BV	1st	Yes	17	—	4.4	0.08	28	28
M	69	1	1	FOLFIRI+BV	1st	Yes	14	—	2.3	0.62	49	24
M	70	1	1	FOLFIRI+BV	2nd	Yes	13	—	1.8	0.20	NA	NA
M	58	0	3	XELOX+BV	1st	Yes	13	—	2.7	0.00^a^	NA	NA
M	73	1	1	FOLFIRI+BV	1st	Yes	13	—	2.8	0.13	65	36
F	70	1	1	FOLFIRI+BV	1st	Yes	13	—	3.2	0.64	NA	NA
F	45	0	1	XELIRI+BV	1st	Yes	13	—	5.2	0.00^a^	NA	NA
M	54	1	1	FOLFIRI+BV	1st	Yes	12	—	1.9	0.25	NA	NA
F	75	1	1	XELIRI+BV	1st	Yes	11	—	5.9	0.07	50	72
F	57	1	1	XELIRI+BV	1st	Yes	10	—	3.4	0.00^a^	NA	NA
F	60	1	1	FOLFIRI+BV	2nd	No	14	—	2.0	0.25	25	64
F	72	1	1	FOLFIRI+BV	2nd	No	10	—	2.9	0.33	NA	NA
F	75	0	1	XELOX+BV	1st	No	10	—	6.3	0.16	NA	NA
F	77	1	3	XELOX+BV	1st	No	8	—	6.5	0.10	47	40
M	71	1	1	XELOX+BV	1st	No	8	—	10.8	0.03	29	72
M	72	0	1	XELIRI+BV	1st	No	10	14	4.5	0.02	26	13
F	52	2	1	XELIRI+BV	1st	No	10	11	11.1	0.02	36	52
M	83	1	1	XEL+BV	1st	No	8	10	3.7	0.10	31	64
M	60	1	1	FOLFOX+BV	2nd	No	7	7	2.4	0.15	NA	NA
F	70	2	1	FOLFOX+BV	2nd	Yes	7	9	7.6	0.39	93	44
F	72	1	1	FOLFOX+BV	1st	No	6	6	3.5	0.08	30	52
M	57	0	2	XELIRI+BV	1st	No	6	16	6.1	0.36	NA	NA
M	77	0	3	XELIRI+BV	1st	No	5	6	12.1	0.19	45	56
M	51	0	2	XELOX+BV	1st	No	4	19	4.4	0.02	NA	NA
F	80	2	2	5-FU/FO+BV	2nd	No	4	4	7.7	0.39	NA	NA

Abbreviations: 5FU/FO=5-fluorouracil/folinic acid; Ang-2=angiopoietin-2 (serum concentration); BV=bevacizumab; ECOG=Eastern Cooperative Oncology Group; F=female; FOLFIRI=5-fluorouracil/folinic acid/irinotecan; FOLFOX=5-fluorouracil/folinic acid/oxaliplatin; HPF=high power field; M=male; Metas.=metastasis (number of organs with metastases); MVD=microvessel density; NA=not analyzed; PC=pericyte coverage; VEGF=vascular endothelial growth factor (serum concentration); XEL=capecitabine; XELIRI=capecitabine/irinotecan; XELOX=capecitabine/oxaliplatin.

a Concentration not measurable.

—, No disease progression or death.
