# Supplementary material for: Identification of serum angiopoietin-2 as a biomarker for clinical outcome of colorectal cancer patients treated with bevacizumab-containing therapy
Source: Br J Cancer. 2010 Oct 5;103(9):1407–14. doi: 10.1038/sj.bjc.6605925 (PMC2990609; doi:10.1038/sj.bjc.6605925)
Supplement: Supplementary Figures [file 6605925x1.ppt]

## Slide 1
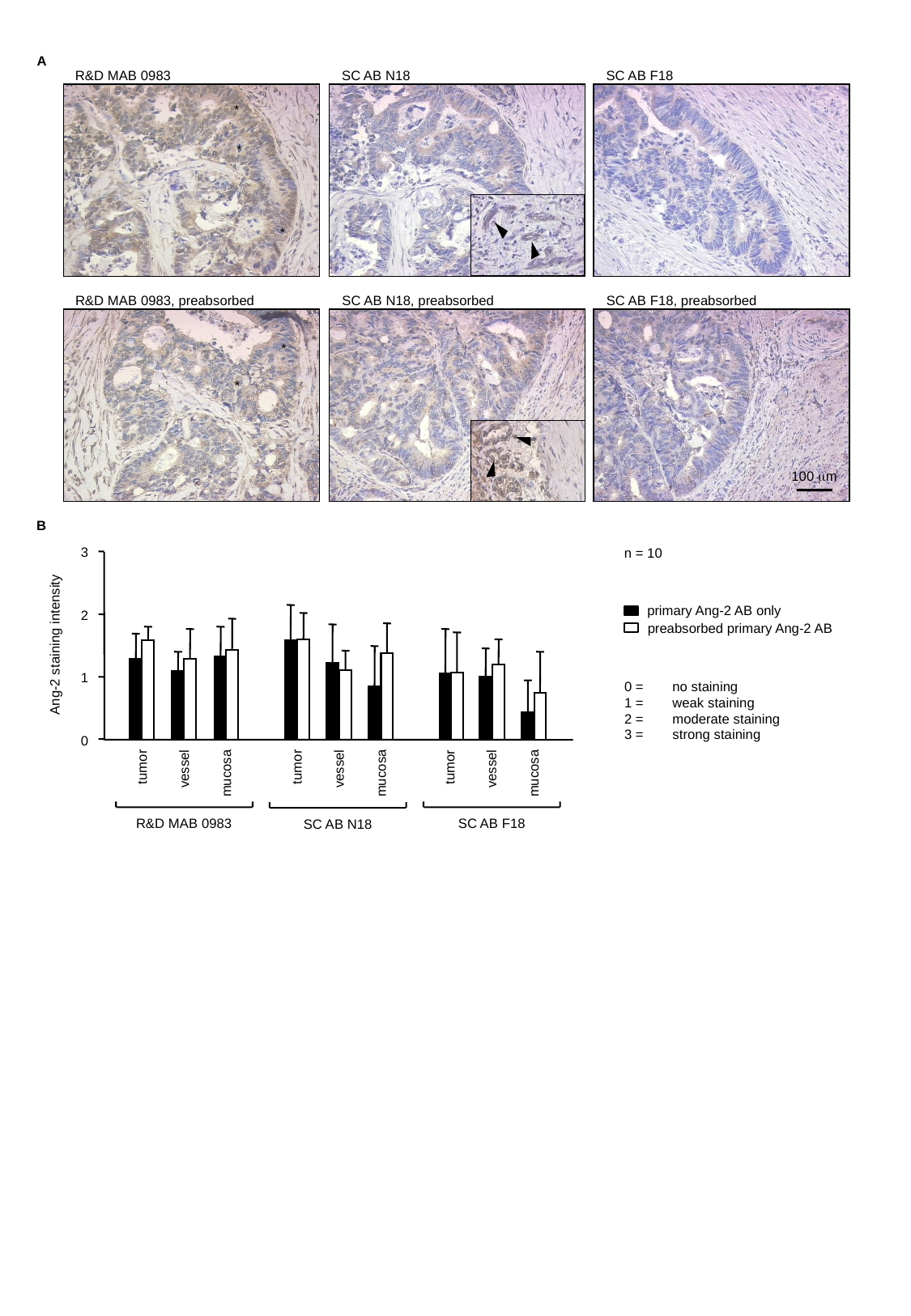

A
R&D MAB 0983
SC AB N18
SC AB F18
*
*
*
SC AB F18, preabsorbed
R&D MAB 0983, preabsorbed
SC AB N18, preabsorbed
*
*
100 μm
B
3
n = 10
primary Ang-2 AB only
2
preabsorbed primary Ang-2 AB
Ang-2 staining intensity
1
0 =	no staining
1 =	weak staining
2 =	moderate staining
3 =	strong staining
0
tumor
tumor
tumor
vessel
vessel
vessel
mucosa
mucosa
mucosa
R&D MAB 0983
SC AB F18
SC AB N18

## Slide 2
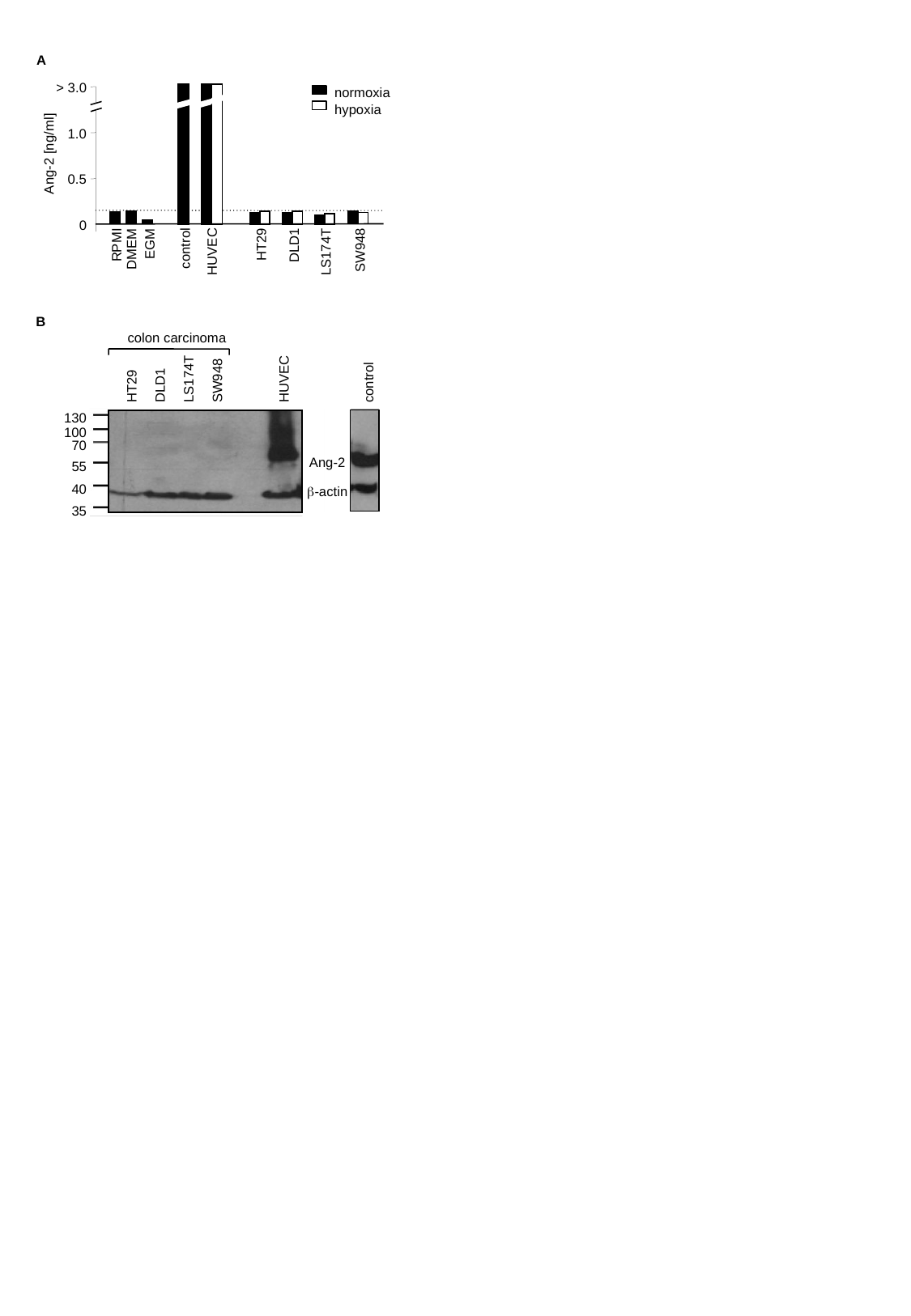

A
normoxia
> 3.0
hypoxia
1.0
Ang-2 [ng/ml]
y
0.5
0
EGM
HT29
RPMI
DLD1
control
DMEM
SW948
HUVEC
LS174T
B
colon carcinoma
HUVEC
LS174T
SW948
control
DLD1
HT29
130
100
70
Ang-2
55
40
β-actin
35
