# Supplementary material for: Identification of serum angiopoietin-2 as a biomarker for clinical outcome of colorectal cancer patients treated with bevacizumab-containing therapy
Source: Br J Cancer. 2010 Oct 5;103(9):1407–14. doi: 10.1038/sj.bjc.6605925 (PMC2990609; doi:10.1038/sj.bjc.6605925)
Supplement: Supplementary Table 1 [file 6605925x2.doc]

*Supplementary Table 1: Treatment outcome by demographics of 34 subjects treated with bevacizumab-containing therapy*

|  |  | OR | p |  | PFS | | |  | OS | | |
| --- | --- | --- | --- | --- | --- | --- | --- | --- | --- | --- | --- |
|  |  |  |  |  | HR | 95%CI | p |  | HR | 95%CI | p |
| Sex |  |  |  |  |  |  |  |  |  |  |  |
| male |  | 61 | 0.73 |  | 1.00 | - | - |  | 1.00 | - | - |
| female |  | 53 |  |  | 1.57 | 0.72 - 3.41 | 0.26 |  | 0.87 | 0.24 - 3.10 | 0.83 |
| Age |  |  |  |  |  |  |  |  |  |  |  |
| < 65 yrs |  | 64 | 0.72 |  | 1.00 | - | - |  | 1.00 | - | - |
| ≥ 65 yrs |  | 53 |  |  | 1.17 | 0.54 - 2.52 | 0.70 |  | 1.34 | 0.38 - 4.77 | 0.65 |
| ECOG |  |  |  |  |  |  |  |  |  |  |  |
| 0 |  | 50 |  |  | 1.00 | - | - |  | 1.00 | - | - |
| 1 |  | 64 | 0.54 |  | 0.76 | 0.32 - 1.82 | 0.54 |  | 0.38 | 0.08 - 1.71 | 0.21 |
| 2 * |  | 33 |  |  | 3.72 | 0.91 - 15.3 | 0.07 |  | 6.70 | 1.26 - 35.8 | 0.03 |
| Treatment |  |  |  |  |  |  |  |  |  |  |  |
| 1^st^ line |  | 58 | 1.00 |  | 1.00 | - | - |  | 1.00 | - | - |
| 2^nd^ line ** |  | 56 |  |  | 1.22 | 0.51 - 2.93 | 0.65 |  | 1.55 | 0.40 - 6.02 | 0.53 |
| Chemotherapy |  |  |  |  |  |  |  |  |  |  |  |
| poly |  | 61 | 0.17 |  | 1.00 | - | - |  | 1.00 | - | - |
| mono *** |  | 0 |  |  | 7.34 | 1.51 - 35.5 | 0.01 |  | 12.9 | 2.34 - 71.3 | 0.00 |
| Metastases |  |  |  |  |  |  |  |  |  |  |  |
| 1 organ |  | 67 | 0.47 |  | 1.00 | - | - |  | 1.00 | - | - |
| 2 organs |  | 50 |  |  | 1.25 | 0.41 - 3.75 | 0.70 |  | 2.90 | 0.69 - 12.2 | 0.15 |
| 3 organs |  | 33 |  |  | 2.97 | 0.84 - 10.5 | 0.09 |  | 1.87 | 0.22 - 12.6 | 0.57 |
| Abbreviations: OR, overall response; PFS, progression-free survival; OS, overall survival; HR, hazard ratio; CI, confidence interval.  * small subgroup of 3 patients only  ** small subgroup of 9 patients only  *** small subgroup of 2 patients only | | | | | | | | | | | |
