# Supplementary material for: Identification of serum angiopoietin-2 as a biomarker for clinical outcome of colorectal cancer patients treated with bevacizumab-containing therapy
Source: Br J Cancer. 2010 Oct 5;103(9):1407–14. doi: 10.1038/sj.bjc.6605925 (PMC2990609; doi:10.1038/sj.bjc.6605925)
Supplement: Supplementary Table 2 [file 6605925x3.doc]

*Supplementary Table 2: Pretherapeutic serum Ang-2 and VEGF concentrations by demographics of 34 subjects treated with bevacizumab-containing therapy*

|  |  | Ang-2 (ng/ml) | p |  | VEGF (ng/ml) | p |  | Remarks |
| --- | --- | --- | --- | --- | --- | --- | --- | --- |
| Sex |  |  |  |  |  |  |  |  |
| male |  | 4.0 | 0.10 |  | 0.24 | 0.51 |  |  |
| female |  | 5.0 |  |  | 0.18 |  |  |  |
| Age |  |  |  |  |  |  |  |  |
| < 65 yrs |  | 3.8 | 0.23 |  | 0.16 | 0.48 |  |  |
| ≥ 65 yrs |  | 4.9 |  |  | 0.23 |  |  |  |
| ECOG |  |  |  |  |  |  |  |  |
| 0 |  | 5.2 | 0.15 |  | 0.14 | 0.17 |  |  |
| 1-2 |  | 4.1 |  |  | 0.22 |  |  |  |
| Treatment |  |  |  |  |  |  |  |  |
| 1^st^ line |  | 4.7 | 0.18 |  | 0.18 | 0.06 |  |  |
| 2^nd^ line |  | 3.7 |  |  | 0.26 |  |  | (subgroup of 9 patients) |
| Chemotherapy |  |  |  |  |  |  |  |  |
| poly |  | 4.3 | 0.27 |  | 0.20 | 0.57 |  |  |
| mono |  | 5.7 |  |  | 0.25 |  |  | (subgroup of 2 patients) |
| Metastases |  |  |  |  |  |  |  |  |
| 1 organ |  | 3.9 | 0.13 |  | 0.21 | 0.92 |  |  |
| 2 organs |  | 5.1 |  |  | 0.22 |  |  |  |
| 3 organs |  | 7.1 |  |  | 0.15 |  |  | (subgroup of 3 patients) |
