# Supplementary material for: Identification of serum angiopoietin-2 as a biomarker for clinical outcome of colorectal cancer patients treated with bevacizumab-containing therapy
Source: Br J Cancer. 2010 Oct 5;103(9):1407–14. doi: 10.1038/sj.bjc.6605925 (PMC2990609; doi:10.1038/sj.bjc.6605925)
Supplement: Supplementary Table 3 [file 6605925x4.doc]

*Supplementary Table 3: Survival by pretherapeutic serum Ang-2 concentrations in 34 subjects receiving bevacizumab-containing therapy as first line (25) or second line (9) treatment*

|  |  | Median PFS  (mo) | p |  | Median OS  (mo) | p |  | Remarks |
| --- | --- | --- | --- | --- | --- | --- | --- | --- |
| 1^st^ Line |  |  |  |  |  |  |  |  |
| low Ang-2 |  | 13.2 | 0.02 |  | not reached | 0.01 |  |  |
| high Ang-2 |  | 8.5 |  |  | 16.2 |  |  |  |
| 2^nd^ Line |  |  |  |  |  |  |  |  |
| low Ang-2 |  | 13.0 | 0.03 |  | not reached | 0.05 |  |  |
| high Ang-2 |  | 7.3 |  |  | 9.4 |  |  | (subgroup of 3 patients) |
