# Supplementary material for: Identification of serum angiopoietin-2 as a biomarker for clinical outcome of colorectal cancer patients treated with bevacizumab-containing therapy
Source: Br J Cancer. 2010 Oct 5;103(9):1407–14. doi: 10.1038/sj.bjc.6605925 (PMC2990609; doi:10.1038/sj.bjc.6605925)
Supplement: Supplementary Figure Legends [file 6605925x5.doc]

*Supplementary Fig. 1:*

**Detection of Ang-2 in CRC by IHC.** **A:** IHC for Ang-2 in paraffin-embedded colon carcinoma tissue using MAB 0983, AB N18 and AB F18 as primary antibodies. Similar immunostaining of tumor cells (asterix) and endothelial cells (insert, arrows) was observed with (lower panels) and without (upper panels) preincubation of primary antibodies with recombinant Ang-2. **B:** Semiquantitative assessment of Ang-2 staining intensity.

*Supplementary Fig. 2:*

**Detection of Ang-2 in CRC by ELISA and WB.** **A:** Quantification of Ang-2 protein by ELISA in supernatants of colon carcinoma cell lines, cell culture medium (negative control) and human umbilical vein endothelial cells (HUVEC) and 293FT cells transfected with Ang-2 expression plasmid (positive control). **B:** Western blot analysis of cytosolic extracts of colon carcinoma cell lines and HUVEC or 293FT cells overexpressing Ang-2.
